# Supplementary material for: Dendrobium officinale Polysaccharides Inhibit 1-Methyl-2-Nitro-1-Nitrosoguanidine Induced Precancerous Lesions of Gastric Cancer in Rats through Regulating Wnt/β-Catenin Pathway and Altering Serum Endogenous Metabolites
Source: Molecules. 2019 Jul 23;24(14):2660. doi: 10.3390/molecules24142660 (PMC6680496; doi:10.3390/molecules24142660)
Supplement: Supplementary file 1 [file molecules-24-02660-s001.pdf]

1. RT-PCR primer sequences

Table S1 RT-PCR primer sequences

Gene name	Forward primer	Reverse primer
β-actin	CCCATCTATGAGGGTTACGC	TTTAATGTCACGCACGATTTC
Wnt2β	GATGGGGCCAATTTCACAGC	AGTTGTGTCATACCCTCGGC
β-catenin	CTTACGGCAATCAGGAAAGC	GACAGACAGCACCTTCAGCA
Gsk3β	ACACCTGCCCTCTTCAACTTTACC	ATTGGTCTGTCCACGGTCTCCA
CyclinD1	GCGTACCCTGACACCAATCT	GCTCCAGAGACAAGAAACG
PCNA	AGGGCTGAAGATAATGCTGATA	CTCATTCATCTCTATGGTCACAG

2. Total serum ion map in positive and negative mode

The peak of each group have great resolution and the baseline is stable which is indicated that the experimental conditions could separate the substances and was suitable for detecting the endogenous substances in rats serum(Figure. S2).

A

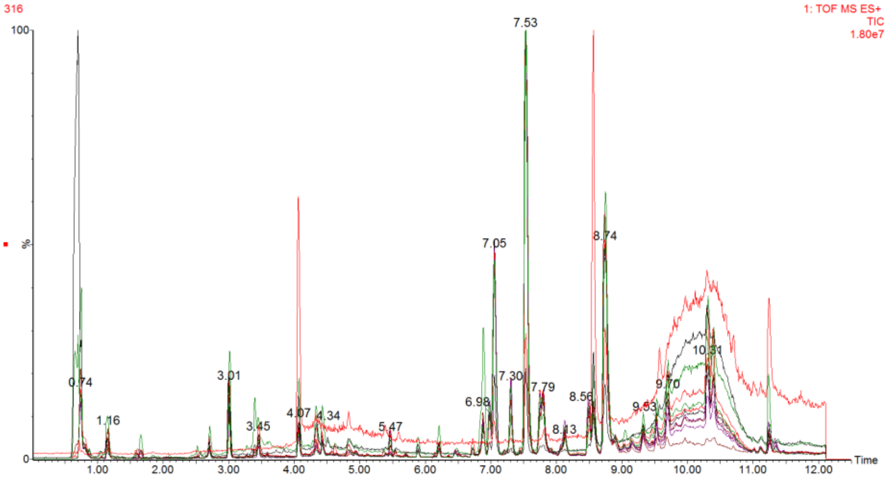

B

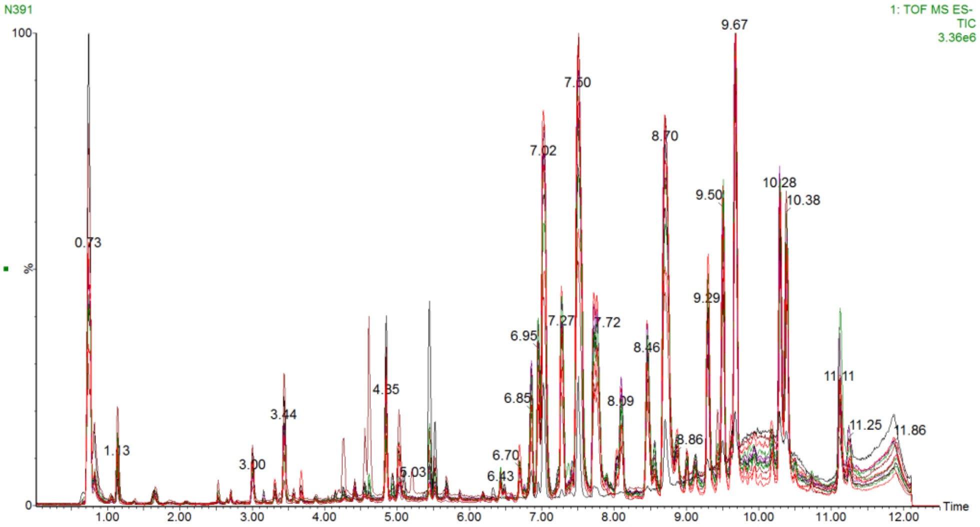

Figure S1. The effect of DOP on the total ion chromatogram (TIC). Results from administering DOP 7 months on PLGC rat model, then collecting the serum to build the available method by UPLC/Q-TOF-MS. The detail UPLC and Q-TOF-MS methods were described in the Materials and Methods. The different color of the ion chromatogram means the one sample from the each group, the result showed that the peak time of sample was the same and indicated that this method will be fit for the all groups. A: the total ion chromatogram on the positive mode; B. the total ion flow chromatogram on the negative mode.

3. The Western blot analysis of other two samples, the result indicated that expression of Wnt2β, β-cetanin, PCNA, CyclinD1 were all upregulated in the PLGC model group than the CTRL group, after treated with DOP, the H-DOP could significantly downregulated the expression of Wnt2β, β-cetanin, PCNA and CyclinD1.

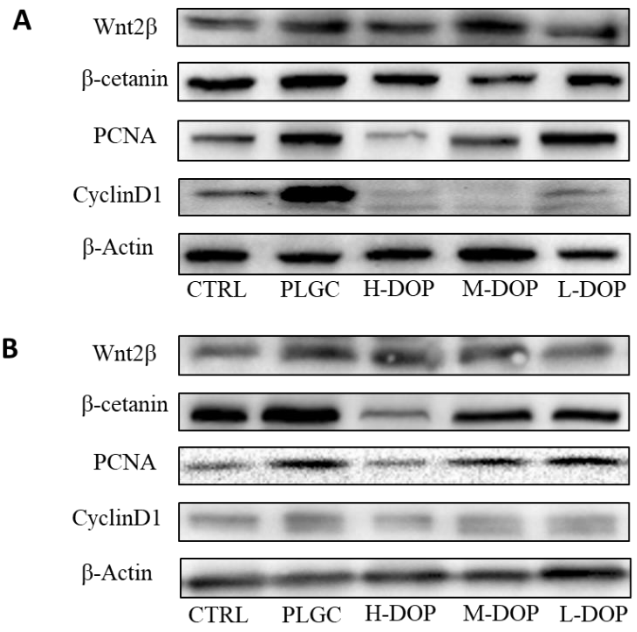

Figure S2. The Western blot analysis of other two samples. (A. the first sample; B the second sample)
